# Assessment of Retinal and Choroidal Measurements in Chinese School-Age Children with Cirrus-HD Optical Coherence Tomography

**DOI:** 10.1371/journal.pone.0158948

**Published:** 2016-07-08

**Authors:** Tao Li, Xiaodong Zhou, Zhi Wang, Jie Zhu, Wenli Shen, Bo Jiang

**Affiliations:** Department of Ophthalmology, Fudan University Jinshan Hospital and, 1508 Longhang Road, Shanghai, China; Charité University Medicine Berlin, GERMANY

## Abstract

**Purpose:**

To evaluate retinal thickness (RT), retinal volume (RV) and choroidal thickness (ChT) in Chinese children using Cirrus-HD optical coherence tomography (OCT), and assess their associations with spherical equivalent (SE), age and gender.

**Methods:**

This was a prospective study that recruited 193 healthy Chinese children (193 eyes) with no ophthalmic disease history between December 2012 and December 2013. RT and RV were acquired using OCT. Subfoveal ChT (SFCT) and ChT1-mm and 2-mm temporal, nasal, superior and inferior to the fovea were measured manually.

**Results:**

RT in the inner temporal and nasal regionsdiffered significantly between refraction groups (both *P*<0.05). Significant differences were also found inSFCT andChT 1- and 2-mm inferior to the fovea (all *P*<0.05). RT differed significantly between males and females in the outer superior region in the emmetropia group (*P<*0.05). ChT differed significantly between males and females 2-mm temporal to the fovea in the emmetropia group (*P*<0.05), and 1-mm temporal to the fovea in the mild myopia group (*P*<0.05). SE correlated positively with RT in the inner temporal (r = 0.230),nasal (r = 0.252) and inferior (r = 0.149) regions (all *P*<0.05). Age correlated positively with foveolar (r = 0.169), total macular (r = 0.202), inner temporal (r = 0.237), inner nasal (r = 0.248), inner superior (r = 0.378) and inner inferior (r = 0.345) region thicknesses, and with RV (r = 0.207)(all *P*<0.05). SE correlated positively with SFCT (r = 0.195), and with ChT1-mm temporal (r = 0.167), 1- and 2-mm nasal (r = 0.144 and r = 0.162), 2-mm superior (r = 0.175), and 1- and 2-mm inferior (r = 0.207 and r = 0.238) to the fovea (all *P*<0.05). Age had no significant association with ChT.

**Conclusions:**

SE, age and gender did not influence macular RT and ChT in most regions, and correlations of RT with age and ChT with SE were weak.

## Introduction

Optical coherence tomography (OCT) can provide precise measurements in vivo, allowing assessment of the retinal nerve fiber layer, retinal thickness (RT), retinal volume (RV) and choroidal thickness (ChT). OCT technology is now used widely in the diagnosis and monitoring of posterior retinal and choroidal pathology in humans. An improved understanding of the characteristics of the normal thicknesses of the retina and choroid in childhood should assist in the diagnosis of abnormalities in these structures that are associated with ocular diseases. Many different ocular diseases, including high myopia [1], inherited retinal disease [2]and glaucoma [3], have been found to be associated with changes in the thicknesses of the retina and choroid.

Successful use of OCT in children has been demonstrated, and its measurements reported to be reliable, accurate and repeatable [4–11]. Recent studies have established that retinal and choroidal thickness in children is influenced by a number of variables, including retinal and choroidal location [7–11] and age [6, 9], while the influence of gender is less clear [11]. Zhang et al. [11] analyzed the macular parameters between genders, and found that the minimum foveal thickness, foveal volume, and average inner ring and temporal outer quadrant macular thicknesses were significantly greater in boys than in girls. However, the values were not compared between different ranges of refractive error.

Cirrus-HD OCT has been reported to provide reliable measurements of retinal and choroidal thickness [12–14]. The purpose of this study was to evaluate RT, RV and ChT in Chinese school children aged 7–15 years using Cirrus-HD OCT, and to examine the associations of these parameters with spherical equivalent (SE), age and gender.

## Materials and Methods

### Study Population

From December 2012 to December 2013, a total of 193 children (193 eyes) were enrolled at the Department of Ophthalmology, Fudan University Jinshan Hospital, Shanghai, China. The inclusion criteria were: 7–15 years of age; Chinese ethnicity; best corrected visual acuity ≥ 1.0; SE ranging between +1 D and -6 D with astigmatism < 1 D; normal intraocular pressure (IOP ≤ 21 mmHg); normal optic nerve head without glaucomatous changes; and no retinal or choroidal abnormalities other than myopic peripapillary atrophy. The exclusion criteria were: a history of retinopathy; a history of prematurity; amblyopia; strabismus; or systemic diseases.

The enrolled children were allocatedinto three groups according to the SE. Emmetropia was defined as a SE of between -0.5 D and +1.0 D; mild myopia as -3 D ≤ SE < -0.5 D; and moderate myopia as -6 D ≤ SE < -3 D.

All procedures conformed to the tenets of the Declaration of Helsinki. This study was approved by the Ethics Committee of Fudan University Jinshan Hospital. Written informed consent was obtained from the children’s parents or guardians.

### Ocular Examination

All participants underwent a complete ophthalmic examination, including visual acuity, slit lamp biomicroscopy, dilated fundus examination with direct ophthalmoscopy, cycloplegic refraction, and IOP measurements. A slit lamp (Suzhou Visual Technology Co., Ltd, Suzhou, China) wasused to examine the anterior segment. The IOP was determined by noncontact tonometry (CT-80; Canon Inc., Tokyo, Japan). Cycloplegia was achieved by topical application of six drops of 0.5% tropicamide(Bausch &Lomb Pharmaceutical Co., Ltd, Shandong, China) at five-minute intervals. Subjective refractive results were obtained 30minutes after the last eye drop was administered.All measurements were made by suitably qualified and experienced professionals (TL, JZ, WLS and BJ).

### OCT Measurements

Macular thickness scanning was performed through dilated pupils (Cirrus-HD OCT 4000; Carl Zeiss Meditec, Inc., Dublin, USA), using a macular cube 512 × 128 scan protocol. This scan protocol generates a cube of data through a 6-mm square grid by acquiring a series of 128 horizontal scan lines comprising 512 A scans. The macular cube scans were performed with the participant’s head fixed on a sustainer, upright and with the eye focusing on an internal fixation target, and in the absence of blinking and eye movement. Once the macula was centered on the live scanning-laser image, a 6.0 × 6.0 mm square of data was captured with signal strengths of at least seven out of ten. An average of the macular cube scans from three measurements was used for subsequent analysis. The refractive power of each participant was compensated for by adjusting the focus knob to a value closest to the spherical value of the eye examined.

**Fig 1:**
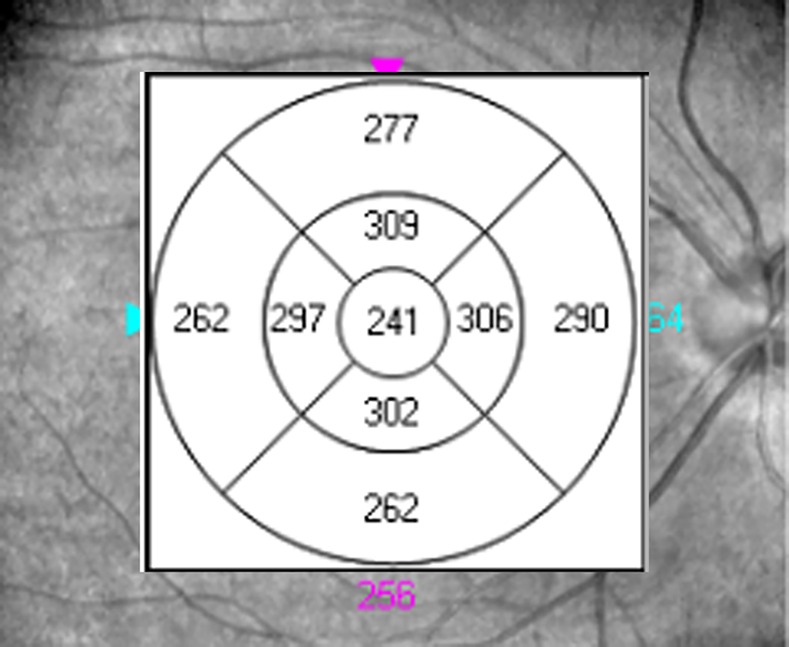
Example of retinal thickness (μm) measurement using Cirrus HD optical coherence tomography.

RT over the macula was determined automatically by an algorithm as the distance between the vitreoretinal interface and the boundary corresponding to the photoreceptor inner-outer segment junction [15]. After completion of the scan, the macula was divided automatically into three concentric regions [11]: the central disc, referred to as the fovea, was a region with a radius of 1mm; and the inner and outer rings had outer radii of 3 and 6 mm, respectively. The inner and outer rings were segmented into four quadrants (superior, nasal, inferior and temporal) by two reticules. Hence, the 6-mm-diameter macula was divided into nine areas arranged in three concentric rings (Fig 1) as defined by the Early Treatment Diabetic Retinopathy Study Group (ETDRS): the foveola, fourinner regions (temporal, nasal, superior and inferior), and fourouter regions (temporal, nasal, superior and inferior). RV was calculated as the total volume of tissue within the cube (6.0 × 6.0 mm) that comprisedthe inner limiting membrane, nerve fiber layer, ganglion cell layer, inner plexiform layer, inner nuclear layer, outer plexiform layer, outer nuclear layer, external limiting membrane, photoreceptor layer and retinal pigment epithelium[16].The mean RT in the9 sectors andthe RV were automatically calculated.

**Fig 2:**
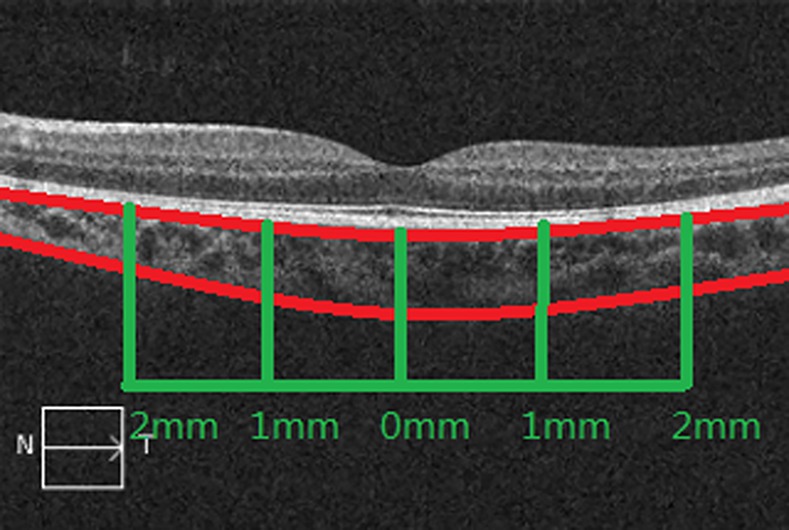
Example of OCT with horizontal scanning. Choroidal thickness was measured at the fovea and 1- and 2-mm temporal, nasal, superior and inferior to the fovea.

ChT was measured from the outer portion of the hyperreflective line corresponding to the retinal pigment epithelium to the inner surface of the sclera [13], using the Cirrus linear measurement tool. ChT was measured at the fovea, and 1-mm and 2-mm temporal, nasal, superior and inferior to the fovea (Fig 2). The mean thickness 1-mm from the fovea was referred to as the parafoveal zone ChT, and the mean thickness 2-mm from the fovea was referred to as the perifoveal zone ChT. For each location, the mean ChT value from three measurements was used for the analysis. All measurements were made by the same examiner (TL or JZ) who was masked to the SE.

### Statistical Analysis

Measurements used for data analysis were obtained only from the right eye of each participant. SE was defined as spherical power plus half-negative cylinder power. All data were analyzed using SPSS version 17.0 software (SPSS Inc., Chicago, IL, USA). The distribution of each parameter was assessed using theKolmogorov-Smirnov test. For normally distributed variables,data are expressed as the mean ± standard deviation, statistical comparisons between groups were made using one-way analysis of variance (ANOVA) with the Bonferroni post-hoc test, and associations were analyzed using Pearson correlation analysis. For parameters not normally distributed, data are expressed as the median (interquartile range),statistical comparisons between groups were made using the Kruskal-Wallis test andpost-hocMann-Whitney U tests, and associations were analyzed using Spearman correlation analysis.The OCT measurements were further analyzed by gender, and intersex differences assessed using the independent-samples *t*-test.The intraclass correlation coefficient (ICC) was estimated to assess the intraexaminer repeatability of the ChT measurements, using a one-way random-effects ANOVA. *P* <0.05 was considered statistically significant.

## Results

The characteristics of the subjects are listed in Table 1. The mean age of the children was 11.5 ± 1.7 years, and 90 (46.6%) were female. There were no significant differences in age, gender or IOP between groups. The Kruskal-Wallis test revealed a significant difference in SE between groups (*P*< 0.001), andsubsequent Mann-Whitney U tests showed that each group differed significantly from the other two groups (all *P*< 0.05).

**Table 1:** Characteristics of the study participants.

Variable	Emmetropia(*n* = 71)	Mild myopia(*n* = 65)	Moderate myopia(*n* = 57)	*P*
Age (y)	11.7 ± 1.7	11.4 ± 1.5	11.5 ± 2.0	0.530*
Gender (M/F)	35/36	32/33	36/21	NA
SE (D)	0(0.50)	-1.50(1.00)^#^	-4.25(1.25)^#^^§^	<0.001^†^
IOP (mmHg)	16.2 ± 2.6	16.7 ± 2.1	17.0 ± 1.9	0.117*

SE, spherical equivalent; IOP, intraocular pressure; NA, no assessment.

*One-way analysis of variance

^†^Kruskal-Wallis test.

^#^*P*< 0.05vs emmetropia group (Mann-Whitney U test)

^§^*P*< 0.05vs mild myopia group (Mann-Whitney U test).

### Retinal Measurements

Mean foveolar thickness and total macular thickness were 240.5 ± 20.0 μm and 280.0 ±13.9 μm, respectively. Mean RV was 10.0 ± 0.5 mm^3^. As illustrated in Table 2, RT differed significantly between the various refraction groups in the inner temporal and nasal regions (*P* = 0.002 and *P*< 0.001, respectively; ANOVA), but not in all the other areas. Post-hoc tests showed that RT in the inner temporal and nasal regions was smaller in the mild myopia and moderate myopia groups than in the emmetropia group (all *P*< 0.05). There were no significant differences in RV between the three refraction groups. RT measurements for the various refraction groups stratified by gender are presented in Table 3. The only significant difference between males and females was for RT in the outer superior region in the emmetropia group (*P* = 0.049).

**Table 2:** Retinal thickness and volume measurements in the various refraction groups.

Parameter	Region	Emmetropia	Mild myopia	Moderate myopia	P*	*P* values for Bonferroni post-hoc test		
		(*n* = 71)	(*n* = 65)	(*n* = 57)		emm vs mild myo	emm vs mod myo	mild myo vs mod myo
Retinal thickness (μm)	Foveola	242.7 ± 24.4	238.8 ± 14.4	239.6 ± 19.4	0.480	NA	NA	NA
	Total macula	282.9 ± 12.5	280.5 ± 13.0	277.1 ± 15.1	0.058	NA	NA	NA
	Inner temporal	311.9 ± 13.8	306.7 ± 13.3	303.0 ± 14.2	0.002	0.030	<0.001	0.145
	Inner nasal	325.3 ± 13.2	320.3 ± 15.7	314.1 ± 14.5	<0.001	0.045	<0.001	0.189
	Inner superior	321.9 ± 20.2	317.8 ± 17.0	314.5 ± 14.9	0.065	NA	NA	NA
	Inner Inferior	317.0 ± 18.9	313.1 ± 15.0	310.0 ± 15.9	0.062	NA	NA	NA
	Outer temporal	269.1 ± 14.8	265.7 ± 10.7	263.3 ± 17.6	0.081	NA	NA	NA
	Outer nasal	302.1 ± 15.3	302.6 ± 15.1	296.7 ± 18.8	0.099	NA	NA	NA
	Outer superior	287.1 ± 17.5	284.7 ± 14.8	283.1 ± 17.3	0.395	NA	NA	NA
	Outer Inferior	273.0 ± 14.8	268.1 ± 14.2	267.3 ± 15.7	0.061	NA	NA	NA
Retinal volume (mm^3^)		10.1 ± 0.4	10.0 ± 0.5	9.9 ± 0.6	0.062	NA	NA	NA

Data are expressed as the mean ± standard deviation.

* One-way analysis of variance (ANOVA); emm: emmetropia group; mild myo: mild myopia group; mod myo: moderate myopia group; NA: not assessed (post-hoc analyses not merited due to non-significant result with the initial ANOVA test).

**Table 3:** Retinal thickness measurements in male and female participants of the various refraction groups.

Parameter	Region	Emmetropia			Mild myopia			Moderate myopia		
		M (*n* = 35)	F (*n* = 36)	*P*	M (*n* = 32)	F (*n* = 33)	*P*	M (*n* = 36)	F (*n* = 21)	*P*
Retinal thickness (μm)	Foveola	240.3 ± 29.9	245.0 ± 17.6	0.426	239.1 ± 14.5	238.5 ± 14.5	0.852	242.6 ± 20.3	234.3 ± 17.0	0.105
	Total macula	280.1 ± 11.9	285.5 ± 12.6	0.067	281.2 ± 12.8	280.0 ± 13.3	0.694	276.5 ± 16.6	278.1 ± 12.4	0.674
	Inner temporal	310.4 ± 14.5	313.3 ± 13.2	0.375	308.7 ± 14.3	304.7 ± 12.2	0.235	303.6 ± 15.6	302.0 ± 11.6	0.660
	Inner nasal	323.7 ± 13.0	326.8 ± 13.5	0.320	322.7 ± 17.2	317.9 ± 13.9	0.227	315.8 ± 15.5	311.1 ± 12.6	0.227
	Inner superior	318.9 ± 21.9	324.7 ± 18.3	0.232	318.8 ± 19.3	316.8 ± 14.6	0.636	316.4 ± 15.7	311.2 ± 13.1	0.186
	Inner inferior	313.3 ± 22.9	320.6 ± 13.3	0.105	313.6 ± 15.5	312.6 ± 14.8	0.781	311.4 ± 16.8	307.6 ± 14.1	0.374
	Outer temporal	267.1 ± 15.2	271.0 ± 14.2	0.271	266.0 ± 12.3	265.5 ± 9.0	0.857	261.3 ± 18.2	266.7 ± 16.3	0.256
	Outer nasal	301.2 ± 11.5	302.9 ± 18.5	0.628	303.8 ± 17.4	301.3 ± 12.7	0.505	297.6 ± 19.9	295.2 ± 17.0	0.625
	Outer superior	283.0 ± 13.3	291.1 ± 20.2	0.049	283.8 ± 15.9	285.6 ± 13.8	0.617	282.3 ± 18.2	284.5 ± 16.1	0.639
	Outer inferior	270.8 ± 13.0	275.1 ± 15.0	0.228	266.0± 14.6	270.2 ± 13.7	0.233	265.5 ± 18.4	270.2 ± 9.1	0.282
Retinal volume (mm^3^)		10.0 ± 0.4	10.2 ± 0.4	0.106	10.0 ± 0.5	10.0 ± 0.5	0.574	9.9 ± 0.6	9.9 ± 0.4	0.853

Data are expressed as the mean ± standard deviation and wereanalyzed using the independent-samples *t*-test.

### Choroidal measurements

The ICC for measurements of ChT was 0.975 (95% confidence interval, 0.966–0.981), indicating high intraexaminer repeatability. The mean subfoveal ChT (SFCT) was 264.2 ± 45.6 μm, which was the greatest value of all the measured locations. As illustrated in Table 4, significant differences between the various refraction groups were found for SFCT and ChT 1- and 2-mm inferior to the fovea (*P* = 0.037, *P* = 0.029 and *P* = 0.005, respectively; Kruskal-Wallis test). Post-hoc analyses (Mann Whitney U tests) showed that SFCT and ChT 1- and 2-mm inferior to the fovea were all significantly greater in the emmetropia group than in the moderate myopia group (*P* = 0.012, *P* = 0.009 and *P* = 0.001, respectively). There were no other significant differences between groups.

**Table 4:** Choroidal thickness measurements in the various refraction groups.

Location relative to fovea	Emmetropia(*n* = 71)	Mild myopia(*n* = 65)	Moderate myopia(*n* = 57)	P*	*P* values for Bonferroni post-hoc test		
					emm vs mild myo	emm vs mod myo	mild myo vs mod myo
SFCT	272.0 ± 43.1	266.7 ± 45.4	251.7 ± 47.1	0.037	0.333	0.012	0.068
1-mm Temporal	263.9 ± 46.0	262.8 ± 41.2	247.0 ± 44.1	0.063	NA	NA	NA
1-mm Nasal	243.0 ± 49.5	225.8 ± 59.6	224.2 ± 51.0	0.082	NA	NA	NA
1-mm Superior	250.2 ± 43.3	246.9 ± 40.7	239.9 ± 47.5	0.411	NA	NA	NA
1-mm Inferior	262.5 ± 44.7	256.2 ± 43.9	241.2 ± 47.5	0.029	0.333	0.009	0.070
2-mm Temporal	257.7 ± 52.2	261.4 ± 46.2	247.1 ± 41.9	0.230	NA	NA	NA
2-mm Nasal	206.1 ± 51.1	194.4 ± 56.5	186.7 ± 50.7	0.113	NA	NA	NA
2-mm Superior	247.8 ± 37.0	245.8 ± 42.2	230.4 ± 52.3	0.059	NA	NA	NA
2-mm Inferior	263.5 ± 46.6	249.5 ± 45.2	236.8 ± 43.3	0.005	0.073	0.001	0.125

Data are expressed as the mean ± standard deviation (μm).

* One-way analysis of variance; emm: emmetropia group; mild myo: mild myopia group; mod myo: moderate myopia group;SFCT: subfoveal choroidal thickness; NA: not assessed (post-hoc analyses not merited due to non-significant result with the initial ANOVA test).

The ChT measurements for the various refraction groups stratified by gender are presented in Table 5. There were significant differences between males and females in ChT 2-mm temporal to the fovea in the emmetropia group (*P* = 0.027), and 1-mm temporal to the fovea in the mild myopia group (*P* = 0.042).

**Table 5:** Choroidal thickness measurements in male and female participants in the various refraction groups.

Location relative to fovea	Emmetropia			Mild myopia			Moderate myopia		
	M (*n* = 35)	F (*n* = 36)	*P*	M (*n* = 32)	F (*n* = 33)	*P*	M (*n* = 36)	F (*n* = 21)	*P*
SFCT	277.5 ± 50.5	266.7 ± 34.3	0.296	259.7 ± 40.9	273.5 ± 49.1	0.223	248.2 ± 53.2	257.7 ± 34.6	0.416
1-mm Temporal	272.7 ± 48.2	255.3 ± 42.8	0.112	252.3 ± 35.3	273.0 ± 44.3	0.042	245.4 ± 46.1	249.9 ± 41.5	0.707
1-mm Nasal	249.7 ± 51.8	236.4 ± 46.9	0.261	218.0 ± 50.3	233.3 ± 67.3	0.301	224.1 ± 52.9	224.4 ± 48.8	0.983
1-mm Superior	257.0 ± 49.4	243.6 ± 35.8	0.196	239.2 ± 41.3	254.4 ± 39.3	0.133	241.2 ± 47.4	237.7 ± 48.8	0.793
1-mm Inferior	271.1 ± 48.6	253.6 ± 39.1	0.086	254.7 ± 39.5	257.6 ± 48.3	0.786	235.9 ± 52.4	250.2 ± 37.3	0.235
2-mm Temporal	271.5 ± 51.5	244.3 ± 49.9	0.027	252.8 ± 42.4	269.7 ± 48.8	0.140	250.1 ± 46.7	242.0 ± 32.4	0.445
2-mm Nasal	212.0 ± 60.6	200.4 ± 39.8	0.346	180.9 ± 45.4	207.5 ± 63.5	0.057	187.2 ± 51.0	185.9 ± 51.6	0.926
2-mm Superior	247.6 ± 40.7	248.0 ± 33.7	0.962	236.6 ± 40.1	254.8 ± 42.9	0.083	231.3 ± 56.3	228.9 ± 45.9	0.858
2-mm Inferior	268.7 ± 49.1	258.3 ± 44.2	0.350	244.8 ± 44.9	253.9 ± 45.8	0.422	230.1 ± 46.6	248.3 ± 34.9	0.101

Data are expressed as the mean ± standard deviation (μm), and were analyzed using the independent-samples *t*-test.SFCT, subfoveal choroidal thickness.

### Associations of the OCT measurements with SE and age

Associations of the macular measurements with SE and age were investigated by correlation analysis (Table 6). SE correlated positively (but weakly) with RT in the inner temporal (r = 0.230, *P* = 0.001), nasal (r = 0.252, *P*< 0.001) and inferior (r = 0.149, *P* = 0.039) regions. Age correlated positively (but weakly) with foveolar thickness (r = 0.169, *P* = 0.019), total macular thickness (r = 0.202, *P* = 0.005), inner region thickness (r = 0.237, *P* = 0.001 for temporal; r = 0.248, *P*< 0.001 for nasal; r = 0.378, *P* < 0.001 for superior; and r = 0.345, *P* < 0.001 for inferior), and RV (r = 0.207, *P* = 0.004).

**Table 6:** Correlation of retinal thickness and volume with spherical equivalent and participant age.

Region	Spherical equivalent		Age	
	r	*P*^‡^	r	*P*^#^
Thickness				
Foveola	0.048	0.511	0.169	0.019
Total macula	0.133	0.064	0.202	0.005
Inner region				
Temporal	0.230	0.001	0.237	0.001
Nasal	0.252	<0.001	0.248	<0.001
Superior	0.136	0.060	0.378	<0.001
Inferior	0.149	0.039	0.345	<0.001
Outer region				
Temporal	0.129	0.074	0.140	0.052
Nasal	0.104	0.150	0.139	0.055
Superior	0.082	0.254	0.066	0.360
Inferior	0.109	0.132	0.107	0.137
Retinal volume	0.134	0.064	0.207	0.004

^‡^ Spearman correlation analysis

^#^Pearson correlation analysis.

**Fig 3:**
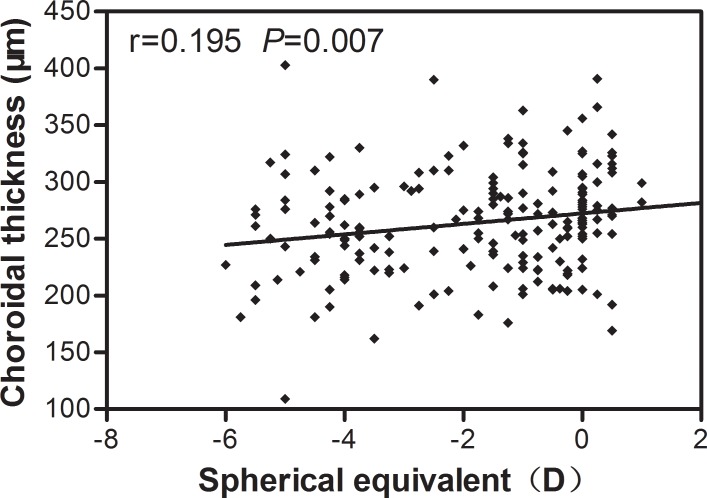
Spearman correlation analysis showed that subfoveal choroidal thickness was weakly positively correlated with spherical equivalent.

**Fig 4:**
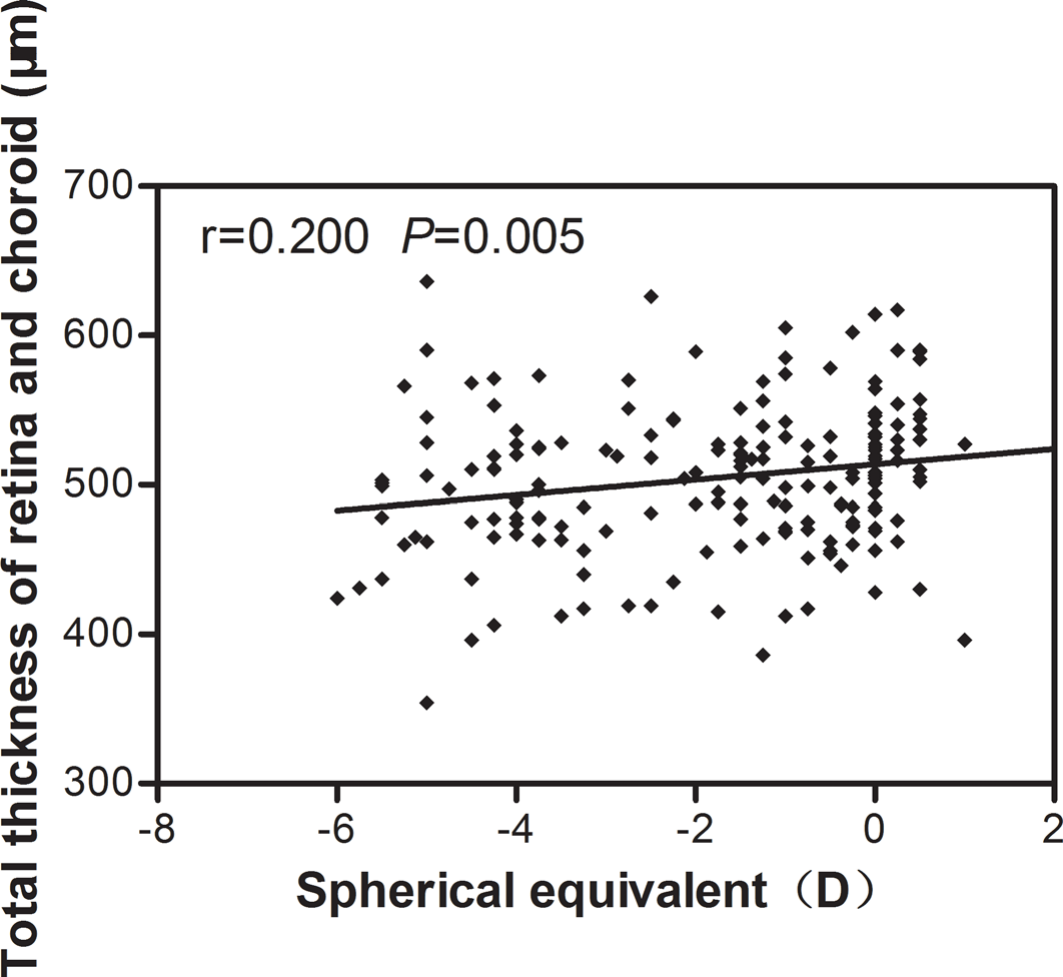
Spearman correlation analysis showed that subfoveal choroidal thickness was weakly positively correlated with spherical equivalent.

Table 7 shows the correlations between SE and ChT measurements at the various locations. SE correlated positively (but weakly) with SFCT (r = 0.195, *P* = 0.007; Fig 3), and with ChT 1-mm temporal (r = 0.167, *P* = 0.020), 1-mm nasal (r = 0.144, *P* = 0.046), 1-mm inferior (r = 0.207, *P* = 0.004), 2-mm nasal (r = 0.162, *P* = 0.024), 2-mm superior (r = 0.175, *P* = 0.015) and 2-mm inferior (r = 0.223, *P* = 0.001) to the fovea. None of the ChT measurements displayed a significant correlation with age. SE correlated positively (but weakly) with the total thickness of the macula retina and choroid (r = 0.200, *P* = 0.005; Fig 4). Foveolar thickness did not correlate significantly with SFCT (r = 0.035, *P* = 0.629).

**Table 7:** Correlation of choroidal thickness with spherical equivalent and participant age.

Location relative to fovea	Spherical equivalent		Age	
	r	*P*^‡^	r	*P*^#^
SFCT	0.195	0.007	0.026	0.717
1-mm Temporal	0.167	0.020	0.096	0.186
1-mm Nasal	0.144	0.046	0.013	0.866
1-mm Superior	0.114	0.114	0.058	0420
1-mm Inferior	0.207	0.004	0.011	0.875
2-mm Temporal	0.085	0.238	0.072	0.318
2-mm Nasal	0.162	0.024	0.019	0.796
2-mm Superior	0.175	0.015	0.046	0.525
2-mm Inferior	0.238	0.001	0.030	0.680

SFCT, subfoveal choroidal thickness.

^‡^ Spearman correlation analysis

^#^Pearson correlation analysis.

## Discussion

In the present study, RT, RV and ChT were measured in Chinese children and their relationships with refraction, age and gender investigated. To the best of our knowledge, this is the first study to analyze the effects of gender on these parameters in various refraction groups. The use of a relatively large sample size of children with a narrow range of SE and a specific age range allowed our study to provide a more detailed examination of whether there are gender differences in RT and ChT in childhood than has been performed previously.

The current study identified the central macula as the thinnest region (240.5 μm at the foveola) followed by the outer ring. The inner ring was thickest due to the presence of a ganglion cell layer, inner nuclear layer and outer plexiform layer. According to previous studies, foveal thickness is greater in adults [17] than in children [11, 18]. Our study found that foveolar thickness, total macular thickness, inner region thickness and RV were positively correlated with age, while outer region thickness was not. A study of Chinese adults revealed that age correlated positively with mean foveal thickness, but negatively with inner and outer macular thicknesses [19]. The conduction of a longitudinal study would be an optimal method for investigating the effects of age on macular thickness and volume.

In the present study, there were no significant differences in macular RT and RV between the various refraction groups (with the exception of the inner nasal and temporal regions). The SE of the enrolled children ranged from +1 D to -6 D, without high myopia. We found that foveolar thickness, total macular thickness and RV did not correlate significantly with SE. Furthermore, RT varied significantly with SE only in the inner nasal and temporal regions, and the correlations were weak. Some studies have reported that retinal thickness is not related to SE, but that the degree of myopia is associated with thinning of most areas of the perifovea [20–22]. However, an amblyopic eye with higher myopia has a greater foveal minimum thickness than the normal fellow eye [10, 23]. Higher myopia is associated with a thinner inner and outer macula, a thicker fovea and a lower RV [4, 14, 24].

Previous studies [11, 17, 25] have documented males as having thicker retinas than females, for a wide range of SE. However, these investigations have not performed subgroup analysis by SE of the effects of gender on RT. In this study, a narrow SE band was used to minimize the confounding effect of SE on macular thickness. We analyzed the influence of gender on RT in the various refraction groups in an attempt to understand whether gender plays an important role in RT changes within a narrow range of SE. In each group, we found no significant differences between males and females for all quadrants, except for the outer superior region in the emmetropia group.

The mean SFCT of 264.2 μm obtained in this study is consistent with a previous study of Chinese adults [26], but smaller than that measured in other countries for both children [8, 9] and adults [27]. These discrepancies can be largely explained by ethnicity differences. Previous studies have provided estimates of mean SFCT in a diversity of populations that range from 261 to 354 μm.

In our study, the ChT at each location was not significantly associated with age. This may be due to the narrow age range of this study sample. Several investigations have examined the effect of age on ChT, with conflicting results. Read et al. [9]found a relatively rapid increase in ChT in early childhood, whereas some studies have documented a significant decrease in the ChT of normal adult eyes with age [13, 26–28]. Ruiz-Moreno et al. [8]suggested that ChT is greater in children than in adults of varying ages because the choroidal layer thickness diminishes with age. Therefore, it seems that ChT increases from early childhood to adolescence, is maintained at a peak for several years, and then exhibits a gradual decrease into older adulthood.

The present study observed that SFCT and ChT 1- and 2-mm inferior to the fovea were significantly different between the emmetropic and moderate myopic groups. Correlation analysis showed that ChT was positively associated with SE, except for the regions in 1-mm superior and 2-mm temporal to the fovea. Our finding of a reduced ChT in more myopic eyes, made using high-resolution frequency domain-OCT, is consistent with the findings of previous studies [26, 29].

Thinning of the choroid may reflect changes in its vascular and connective tissue structure. Histologically, the choroid is formed mainly by blood vessels, and thus reduced thickness in this structure represents diminished blood supply [30]. The SE-dependent decrease in ChT may be related to the progression of degenerative myopia and the loss of choroidal tissue.

We also analyzed the influence of gender on ChT within a narrow range of SE. A significant difference in ChT between genders was found in the region 2-mm temporal to the fovea in the emmetropia group, and 1-mm temporal to the fovea in the mild myopia group; the vast majority of regions analyzed in the three refraction groups showed no significant differences in ChT between males and females. Ding et al. [26] found no significant difference in SFCT between genders, but SFCT tended to be slightly greater in males than in females. Li et al. [31] reported that SFCT in 93 Danish university students was 62.2 μm thicker in men than in women. Osmanbasoglu et al. [32] concluded the reverse, namely that SFCT in healthy emmetropic subjects was slightly greater in females. These discrepancies may be due to differences in ethnicity and age.

One limitation of the present study is that the measurements of ChT were performed manually, and automated software will be required for a more objective evaluation. Nonetheless, Ikuno et al. [13] found that Cirrus OCT with a manual segmentation technique had high reliability and reproducibility when measuring ChT changes in healthy highly myopic eyes. Furthermore, the ICC in the present study indicated that ChT measurements were made with high repeatability, indicating that the manual segmentation technique provides reliable data; this would be consistent with the study of Ikuno et al. [13].

A second limitation is that it cannot be excluded that some of the significant results may have been statistical artifacts due to non-correction for multiple tests over all comparisons and correlations. One approach to dealing with this issue is to use Bonferroni adjustment of the *P* values to take into account the total number of statistical analyses performed on each dataset, and to quote both corrected and uncorrected *P* values. However, the use of Bonferroni adjustments is considered by many authors to be too conservative: although Bonferroni adjustment reduces the chances of a type I statistical error, it also increases the chances of a Type II error (the probability of accepting the null hypothesis when the alternative is true). In addition, the presentation of two different *P* values for each statistical test can complicate rather than simplify the presentation and interpretation of the results. Some researchers advocate the reporting of unadjusted *P* values, but highlighting the potential issue of using uncorrected *P* values to enable the reader to reach a reasonable conclusion [33]. This is the approach that has been used in the present study. Since each RT and ChT dataset in the present study was subjected to four statistical analyses, the Bonferroni-adjusted *P* value for statistical significance would be 0.05/4 = 0.0125. If Bonferroni-adjusted *P* values were used for analysis of the results, the following comparisons would be re-interpreted from significant to non-significant: inner temporal and inner nasal RT between the emmetropia and mild myopia groups (Table 2); outer superior RT between males and females in the emmetropia group (Table 3); ChT 2-mm temporal to the fovea between males and females in the emmetropia group and ChT 1-mm temporal to the fovea between males and females in the mild myopia group (Table 5); correlation of RT with SE in the inner inferior region and correlation of foveolar thickness with age (Table 6); and correlation of ChT with SE in the 1-mm temporal and nasal and 2-mm nasal and superior regions (Table 7). However, it should be stressed that interpreting the data on the basis of Bonferroni-adjusted rather than unadjusted *P* values does not alter the major conclusions of the study that RT and ChT in the vast majority of regions did not differ between refraction groups or between genders, and that any correlation between RT and age or ChT and SE was weak.

In conclusion, this is the first study to analyze the effect of gender on macular RT, RV and ChT within a narrow range of SE. In most regions analyzed, SE, age and gender did not exert a notable influence on macular RT and ChT, and any correlations of RT with age and ChT with SE were weak. Future studies to determine the influence of gender on RT and ChT will help to improve our understanding of changes in these parameters in children with ocular diseases that affect the macula.

## Supporting Information

S1 File Raw Data. The raw data of all parameters for the individual subjects.
(XLS)
